# The Peter Watson Memorial Lecture “Vision for the World”

**DOI:** 10.1038/s41433-022-02045-y

**Published:** 2022-04-08

**Authors:** Hugh R. Taylor

**Affiliations:** Indigenous Eye Health, Melbourne School of Population and Global Health, The University of Melbourne, Melbourne, Vic Australia

**Keywords:** Scientific community, Health occupations, Public health

## Abstract

In the 1990s attention was drawn to the huge global problem of blindness and vision loss; most of which was unnecessary, being preventable or treatable. This led to the global initiative, Vision 2020. Over the last 30 years a lot of progress has been made in developing and implementing eye care programmes to address this and particularly to reach underserved populations. In 2019 the World Health Organisation produced the World Report on Vision that sets a clear pathway to develop Integrated Person-centred Eye Care. Indicators have been developed to track progress and national governments are to report on their progress. Data on eye health and vision loss have been collected from multiple population-based studies and analysed by the Vision Loss Expert Group. These data show that although the prevalence rates of vision loss and blindness are decreasing around the world, the actual number of people affected is slowly increasing. This is due to both population growth and the aging of the population. To provide the equity in eye care that is required, attention needs to be paid to integrating eye care into primary care and linking it with other specialist services. An important step is the training and development of coordinated eye care teams that are resourced to meet their population-based needs and to monitor the progress being made.

## Introduction

### Peter Watson

It was a great honour to be invited to give the inaugural Peter Watson Memorial Lecture. Professor Peter Watson had an illustrious career [1]. He was a staff member at Addenbrookes Hospital, including being head of their Eye Department. He was also head of the Scleritis Clinic at Moorfields. His research interests included glaucoma and scleritis. His textbook “Sclera and Systemic Diseases” went through multiple editions. He founded the Cambridge Ophthalmology Symposium and served on many professional bodies. He was the President of the Academia Ophthalmology Internationalis (AOI) and a member of the International Council of Ophthalmology (ICO) for some seventeen years during which he started the ICO Assessment or the ICO Exams [2]. The ICO created the Peter Watson Award for the top candidate in the ICO Examinations to honour Peter and the role he played.

Over the years I got to know Peter well and particularly when we started working together on the ICO. In 2006 I spent an enjoyable sabbatical in Cambridge during which I came to know Peter and Anne very well. Peter loved his garden and his tennis, but most of all he loved having fun.

### Global issues of vision loss

In 1994 the International Agency for the Prevention of Blindness (IAPB) and the World Health Organisation (WHO) estimated that there about 45 million people blind in the world [3]. About 60% of that blindness was due to cataract or uncorrected refractive error and that could be fixed right away if the services and care were available [4]. Three conditions: trachoma, vitamin A deficiency and onchocerciasis formed about 15% and were public health problems and needed a public health approach. Another 15% of blindness was caused by diabetic retinopathy and glaucoma, that were long-term chronic diseases that are hard to treat. The remaining 10% was due to AMD and other causes of blindness for which there was no way to treat or prevent at that time.

It was estimated that the number of people blind would double by 2020 to about 90 million if things did not change [4]. However, if we did what we knew how to do, blindness could be reduced by over 70% and by 2020 there would be 25 million people blind. This led to WHO and IAPB launching “Vision 2020: The Right to Sight” in 1999. This was a three-way partnership between WHO, IAPB, and the national partners and national governments. Together they were to develop national Vision 2020 plans and improve the provision of eye care.

“Vision 2020 the Right to Sight” had the goal of the elimination of avoidable blindness by the year 2020, in order to give all people in the world the right to sight [4]. It has three components: effective disease control looking at cataract, trachoma, onchocerciasis, childhood blindness, and refractive error and low vision; and human resource development; and infrastructure development. With the work inspired by Vision 2020 between 1990 and 2010 the prevalence rate of both blindness and moderate to severe vision loss in each subregion dropped quite markedly, although the actual number of people who are blind increased slightly [5].

The prevalence rate of moderate severe visual impairment from cataract had dropped 28% in this time, but the number of people affected went up 33%. The prevalence of blindness from cataract dropped by 39%, although the number blind had increased by 16% [6]. The cataract surgery rate in India for example had increased sixfold over a decade [7], and there was a similar change in China [8]. So there was a great improvement in service delivery, but there was still a long way to go.

Then in 2013 the WHO World Health Assembly put together a Global Action Plan for Universal Eye Care with a target for all the member states to reduce avoidable visual impairment by 25% by 2019 [9]. This differed from Vision 2020 that had aimed at the elimination of avoidable blindness. WHO had broadened the remit to vision loss, that included vision impairment and blindness. They introduced indicators the member states were expected to report against to WHO in 2017 and 2020. These indicators included; the prevalence and causes of visual impairment, the cataract surgery rate and cataract surgery coverage rate, and the number of eye care personnel by each category.

In 2015 the United Nations released the 17 Sustainable Development Goals (SDG) that covered a wide range of activities [10]. Eyecare was related to a number of goals; 3-good health and wellbeing, but also 1-Poverty, 2-Zero hunger, 6-Clean water and sanitation, 10-Reducing inequalities, and 17-Partnerships are also key. In the health goals, the real goal was 3.8 Universal Health Coverage that included eyecare for all. The Lancet Global Health Commission on Global Eye Health showed that every one of the 17 SDGs were impacted by people who had poor vision [11].

The idea of Universal Health Coverage was to extend the population who were covered by health insurance or government services, to expand the range of services provided and integrate eye care, and to reduce the cost and to make eye care affordable [9]. The two eye care indicators WHO introduced for Universal Health Coverage for eyecare were the effective cataract surgery coverage rate and the effective refractive error coverage rate. These were defined as percentage of people who can see 6/18 or better after treatment [12].

The percentage of people who have cataract surgery and who need or should have cataract surgery varies greatly from one country to another. Also the number who did not achieve good vision afterwards varies greatly and in some of the areas a third or a half are still legally blind after cataract surgery [13]. Overall, Ramke found after surgery that 54% of people can see 6/18, but the remaining 46% see less than that and 18% are still blind. In Australia 89% could see at 6/18 or better after surgery but still nearly 2% were legally blind [14]. Why can’t people see after surgery? Often the cause will be the use of an inappropriate intraocular lens through the lack of measurement or range limitation, sometimes uncorrected astigmatism, but it could also be due to poor surgical technique, undetected pre-existing disease, or the lack of correction.

The next big step in global blindness work was the WHO World Report on Vision in 2019 [15]. This report showed that there were at least a billion people who had visual impairment that should be addressed and had not yet been addressed. Moreover, there were another 1.2 billion people who had visual impairment, although their current presenting vision may seem normal because they were using glasses or contact lenses. These people also need regular and ongoing eye care but are often forgotten when planning eye service needs. Without their spectacles or contact lenses they had a visual disability and need an aid to see properly. This is important in the planning of eye services because of the number of people who have an eye problem or need a change in glasses.

The World Report of Vision called for eye care to be an integral part of Universal Health Coverage, and the implementation of Integrated People-centred Eye Care [15]. They recognised the need for health systems research, the monitoring and evaluation of progress, and raising awareness about eye care to encourage and empower people and communities. Integrated People-centred Eye Care was based around empowering communities, re-orienting the model of care to focus on the patient’s journey, with the co-ordination of services within and across sectors, so that people can move safely from primary to secondary or tertiary care if needed. Also, to have integration across sectors, so that for example people with diabetes could receive eye care in a coordinated and integrated way.

### The global burden of vison loss

Professor Rupert Bourne, an eminent ophthalmologist from Cambridge, brought together a group of over 100 ophthalmologists and optometrists from around the world to form the Vision Loss Expert Group (VLEG) [16]. They work closely with the Global Burden of Disease Group (GBD), IAPB and WHO, and are supported by several NGOs. The VLEG has performed systematic reviews of the published and unpublished eye surveys from 1980 that are population-based and have a defined methodology. They also provide data to the GBD, who assess the impact of different conditions, including vision loss, on people’s health and wellbeing.

The global database currently has 528 population-based studies [16]. Many of these studies come from ‘RAABs’ or the Rapid Assessment of Avoidable Blindness, a tool developed by the International Centre for Eye Health in London that provides a quick assessment of visual loss in people over the age of 50 [17]. The other studies were more detailed population-based surveys. The more comprehensive studies tend to come from a limited number of countries and many countries have very few studies. RAABs tend to be done mainly in the low- and middle-income countries. These data are available as the Vision Atlas on IAPB website [18]. This is a wonderful resource that is updated regularly and can give information by country, by cause, by age and so forth.

In 2020 there were 42 million people blind, less than 3/60, with a further 257 million people with moderate severe vision impairment, vision less than 6/18 to 3/60 [16]. Another 295 million people had mild vision loss, less than 6/12 but better than 6/18. These data are all for presenting vision in the better eye. Further, 507 million people had near vision impairment or presbyopia.

The rates of blindness and moderate severe visual impairment increases dramatically in age, so nearly 30% will have moderate to severe vision loss in their 90 s and almost 10% will be blind in their 90 s [16]. Women have a higher rate of vision loss in every age group. The prevalence rate of moderate visual impairment has stayed about the same over time, but blindness prevalence rates dropped significantly. Presbyopia rates remained about the same. However, the actual number of people affected in each of these categories have increased progressively each year over the last 30 years. So although the prevalence rates drop, the actual number of people affected have increased due to both population ageing and Population growth. Although we know what to do for most causes of vision loss, and when we do it, it works well; we need to do far more of it.

The largest cause of moderate vision loss is refractive error, but cataract is also important [19]. The leading cause of blindness is cataract. The other conditions, diabetic retinopathy, glaucoma and AMD, although very important in the high-income countries, are less of a problem at the global level. With age-adjustment, women have a higher rate of blindness from each cause than men except for glaucoma where the rate is higher in men.

There is marked regional variation in the causes of blindness [19]. In Southeast Asian countries, cataract is clearly the major cause. In high-income countries, age-related macular degeneration and glaucoma are increasingly important.

Overall, the prevalence rate of blindness is dropping across all regions [16]. However, the prevalence of blindness from diabetic retinopathy is steadily increasing in Sub Saharan Africa, South and Southeast Asia and Oceania. While one can look at the provision of cataract surgery or glasses, the screening for and treatment of diabetic retinopathy is important and a key factor in Integrated Person-centred Eye Care [15]. However, the number of people affected by vision loss and blindness over the next 30 years will progressively increase if there is not a major change in the way eye care services are provided [20].

There are some real success stories about reducing vision loss too from some conditions, such as trachoma. The WHO has verified 13 countries as having eliminated blinding trachoma and before COVID-19 had plans for 20 more by 2023 [21]. The number of people at risk of trachoma has dropped from 1.3 billion in 2002 to 142 million in 2019. Similarly, the number affected by onchocerciasis have dropped [22]. Onchocerciasis has been eliminated from a number of countries in Central America and it has been dramatically reduced in most of the African countries. The work on onchocerciasis is really being facilitated by MSD, providing ivermectin. Almost 200 million doses a year are being distributed with this extraordinary donation programme.

### The impact of vision loss

A Disability Adjusted Life Year (DALY) is the measure of the overall disease burden expressed as the cumulative number of years lost due to ill health, disability, or early death [23]. A DALY is a combination of the years of life lived with a disability and the years of life lost because of early death. Each cause of ill-health or disability is given a disability weight, perfect health is weighted as ‘0’, and death in ‘1’. A definition used for blindness is someone who is completely blind, which causes a great difficulty in daily activities, worry, anxiety and great difficulty going outside a home without assistance. The early estimates of the disability weight for blindness were about 0.6, so having a severe impact [24]. The GBD undertook a large worldwide survey to recalibrate disability weights [25]. The questions were generally framed “who is less healthy, somebody who is X (say blind) or somebody who has Y (for example neck pain)?” Many responded that blind people were perfectly healthy, they just can’t see, and so the disability weight for blindness was reduced to 0.195. Previous questions had been couched as “who is more disabled” rather than “who is less healthy”. WHO subsequently relooked at this and revised this weight for blindness to be 0.338 [23].

The disability weight given to blindness or vision loss has a very big impact on the overall ranking of the disability attributable to vision loss. For those aged 50 to 74, vision loss rated 19th in the GBD ranking of disability and 15^th^ for those over 75 [26]. On the other hand hearing loss rates 9^th^ for those between 50 to 74 years and 10^th^ for those over 75. When vision loss and hearing loss are combined as sensory loss they rate 6^th^ and 8^th^ respectively, but are still well below lung cancer or Alzheimer’s Disease. The two most leading causes of DALYs are ischaemic heart disease and stroke. Again, when one’s struggling for government funds attention to develop services and resources, the disability weight and ranking are a very important advocacy tools.

### Eye personnel and services

The Lancet Commission on Global Eye Health examined the correlation between the number of ophthalmologists and optometrists per million of a population and the amount of blindness [20]. With many ophthalmologists and optometrists in the high income countries the prevalence of blindness was much lower than in the low- and middle-income countries with fewer eye care providers.

So what is needed to have proper Integrated Person-centred Eye Care? Clearly, we need to train more ophthalmologists. That is critical, but that’s not the whole solution. We need to train eye care teams and they need to be trained to be able to meet the population-based needs of their communities. We need to make sure that comprehensive eye care is integrated into the healthcare system, so we are not working in isolation. We also need to make sure that community level primary eyecare is properly integrated into primary healthcare so that primary care staff do check vision and diabetic retinopathy, but they must have a clear referral pathway for those who need it. We need to make sure that people who are trained have the equipment, infrastructure and support they need to be able to provide the eye care they have been trained to give. They also need to have continuing support, guidance, monitoring and professional development.

The ICO and now the Ophthalmology Foundation play an important role in enhancing education with the ICO Exams, the International Fellowships, Resident Programme Directors courses, and other continuing medical education or continuing professional development programmes [27–29]. As mentioned above, Peter was very aware of the need to improve education and started the ICO Examinations in 1995 [2]. More than 50,000 ophthalmology trainees have taken those exams over the years that have been held in 84 countries. Peter was followed by David Taylor, Simon Keightley and then Clare Davey. This is a very fitting way to remember the legacy of Peter Watson, a man who made so many meaningful contributions.

In summary, there has been some real progress globally in tackling blindness and vision loss. The prevalence of blindness and vision loss have been greatly reduced, however, the number of people blind or visually impaired continues to rise with population growth and increasing ageing. The Integrated Person-centred Eye Care defined in WHO’s World Vision Report shows a clear path ahead that we need to commit to and follow. It is important to monitor and evaluate the progress being made; ‘If it’s not counted, it’s not done’ and ‘If it’s not monitored, it can’t be managed’. We also need to train more eyecare practitioners and teams at all levels of eyecare. By doing this, we can eliminate avoidable vision loss and bring equity in eye health.

## Summary

### What is known about this topic

- Vision loss and blindness have a huge impact on the quality of life and the vast majority of blindness and vision loss occurs in low and middle income countries.
- The rate of blindness increased dramatically with age.
- Over the last 20 years Vision 2020 and the global efforts it facilitated have led to a real decrease in the prevalence rates of blindness, although aging and the increasing populations has meant that the actual number of people blind has increased some.

### What this study adds

- We now understand how eye health impacts on, or is impacted by, each of the UN’s 17 Sustainable Development Goals.
- We know how to prevent or treat most of the causes of blindness and vision loss.
- What is needed now is a concerted effort in each country to implement the WHO recommended integrated people-centred eye care and for eye care providers to work effectively in teams.
